# Differential urinary glycoproteome analysis of type 2 diabetic nephropathy using 2D-LC–MS/MS and iTRAQ quantification

**DOI:** 10.1186/s12967-015-0712-9

**Published:** 2015-11-25

**Authors:** Zhengguang Guo, Xuejiao Liu, Menglin Li, Chen Shao, Jianling Tao, Wei Sun, Mingxi Li

**Affiliations:** Core Facility of Instrument, Institute of Basic Medical Sciences, Chinese Academy of Medical Sciences, School of Basic Medicine, Peking Union Medical College, 5 Dong Dan San Tiao, Beijing, 100005 China; Department of Nephrology, Peking Union Medical College Hospital, Peking Union Medical College, Chinese Academy of Medical Sciences, No. 1 Shuaifuyan, Wangfujing Street, Beijing, China; National Key Laboratory of Medical Molecular Biology, Department of Physiology and Pathophysiology, Institute of Basic Medical Sciences, Chinese Academy of Medical Sciences, School of Basic Medicine, Peking Union Medical College, 5 Dong Dan San Tiao, Beijing, 100005 China; The Center for Biomedical Information, Institute of Basic Medical Sciences, Chinese Academy of Medical Sciences, School of Basic Medicine, Peking Union Medical College, 5 Dong Dan San Tiao, Beijing, 100005 China

**Keywords:** Diabetic nephropathy, Glycoproteomics, Biomarker

## Abstract

**Background:**

Diabetic nephropathy (DN) is the leading cause of chronic kidney failure and end-stage kidney disease. More accurate and non-invasive test for the diagnosis and monitoring the progression of DN is urgently needed for the better care of such patients.

**Methods:**

In this study we utilized urinary glycoproteome to discover the differential proteins during the course of type 2 DN. The urinary glycoproteins from normal controls, normalbuminuira, microalbuminura, and macroalbuminuria patients were enriched by concanavalin A (ConA) and analyzed by 2DLC/MS/MS and isobaric tags for relative and absolute quantitation quantification.

**Results:**

A total of 478 proteins were identified and 408 were annotated as N-linked glycoproteins. A total of 72, 107 and 123 differential proteins were identified in normalbuminuria, microalbuminuria and macroalbuminuria, respectively. By bioinformatics analysis, in normalbuminruia state, cell proliferation and cell movement were activated, which might reflect the compensatory phase during the disease development. In micro- and macro-albuminuria, cell death and apoptosis was activated, which might reflect the de-compensatory phase. Pathway analysis showed acute phase proteins, the member of high density lipoprotein and low density lipoprotein proteins were changed, indicating the role of the inflammatory response and lipid metabolism abnormality in the pathogenesis of DN. Six selected differential proteins were validated by Western Blot. Alpha-1-antitrypsin (SERPINA1) and Ceruloplasmin are the two markers with excellent area under curve values (0.929 and 1.000 respectively) to distinguish the microalbuminuria and normalbuminuria. For the first time, we found pro-epidermal growth factor and prolactin-inducible protein were decreased in macroalbuminuria stage, which might reflect the inhibition of cell viability and the activation of cell death in kidney.

**Conclusions:**

Above data indicated that urinary glycoproteome could be useful to distinguish the differences in protein profiles in different stages in DN, which will help better individualized care of patients in DN.

**Electronic supplementary material:**

The online version of this article (doi:10.1186/s12967-015-0712-9) contains supplementary material, which is available to authorized users.

## Background

As a major microangiopathy complication of diabetes mellitus (DM), diabetic nephropathy (DN) is the leading cause of chronic kidney failure and end-stage kidney disease (ESRD). 36.9 % of all ESRD patients in United States are due to diabetic nephropathy. According to the classification of American Diabetes Association [1], DN is divided into three stages, incipient nephropathy (microalbuminuria), clinical diabetic nephropathy (macroalbuminuria) and ESRD. To diagnose those DN patients in its early stage can effectively prevent or delay the progression to ESRD [2]. Renal biopsy is a useful way to definitive diagnosis. However, renal biopsy is an invasive method which has clinical risks, including massive bleeding, pain, infection and arteriovenous fistula [3]. Also, some patients have such bleeding disorder and some other comorbidity as contraindications for renal biopsy [4]. A simple, accurate and non-invasive test was urgently needed for early diagnosis of DN and/or monitoring its progression [5]. Currently, the presence of microalbuminuria is widely accepted as the sign of onset of DN [6]. However, patients with cardiovascular disease [7, 8], hypertension, and inflammation [2] can also present microalbuminuria. Meanwhile, many patients who already have advanced renal histopathological changes are normoalbuminura [9–11]. As many as 55–70 % microalbuminuria patients do not progress to proteinuria within 10 years, while ~40 % of those who are ultimately at risk of progression to proteinuria are constantly normoalbuminura [6]. This calls for more accurate biomarkers in clinical research and its application.

The urinary proteome can reflect the changes of urinary system, thus urine is a suitable source for biomarker discovery for kidney diseases. As early as 2005, Kumar et al. [12] used two-dimension differential in-gel electrophoresis (2D-DIGE) method to discover biomarkers of DN in urine, identified and validated alpha-1-antitrypsinin DN patients. Until now, many publications have reported the differential proteins in the urine of DN using various proteomic methods [5, 13–31] and a total of more than 200 differential proteins have been found, and some differential proteins had been validated using immunological methods or target proteomic method [14, 16, 18, 19, 28–31]. Above results indicated that urinary proteome might be used to discover DN biomarkers. However, due to the high abundance suppression by albumin in DN urinary proteome, it was difficult to identify the low abundance proteins.

Glycoproteins are involved in many important biological functions, such as cell attachment, regulation of development, immune response, signal transduction, protein folding and hemostasis [32]. More than half of all proteins are thought to be glycoproteins, and the quality and quantity of glycoproteins will change along with the physiological and pathological processes [33]. Because urinary glycoproteome might reflect the functions of kidney and urinary tracts [33], it had been successfully used to discover the biomarkers for bladder cancer [34], chronic kidney disease (CKD) [35]. It was also used to distinguish Adriamycin nephropathy from Thy1.1 glomerulonephritis [36]. Therefore, urinary glycoproteome should become another useful approach for biomarker discovery of DN.

**Fig. 1:**
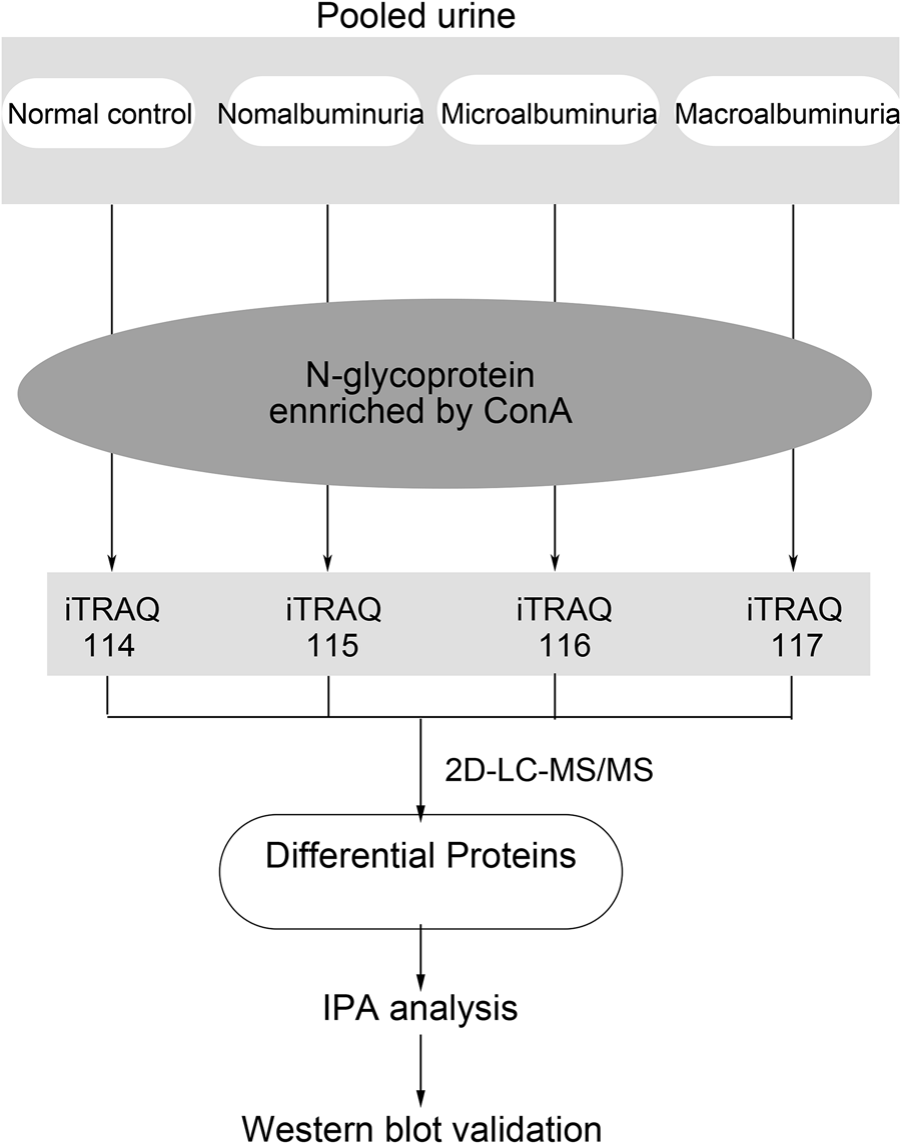
Work flow of differential urinary glycoproteome analysis

In this study, urinary glycoproteome was used to discover the biomarkers in early stage and its progression monitor of DN. The urinary proteins from normal controls, normalbuminuria, microalbuminura, and macroalbuminuria were collected respectively. The urinary N-linked glycoproteins were enriched by concanavalin A (ConA), then labeled by 4-plex isobaric tags for relative and absolute quantitation (iTRAQ) regents, and analyzed by 2D-LC MS/MS. The differential proteins were functionally annotated by Ingenuity Pathway Analysis (IPA). Furthermore, selected differential proteins were validated in individual samples by Western blot (Fig. 1) and the sensitivity and specificity were evaluated.

## Methods

### Reagents and instruments

ConA agarose, HPLC grade acetonitrile (ACN) and formic acid, trifluoroacetic acid, ammonium bicarbonate, iodoacetamide (IAA), and dithiothreitol (DTT) were purchased from Sigma (St. Louis, MO, USA). Sequencing grade trypsin was purchased from Promega (Madison, WI, USA). The 4-plex iTRAQ regents were purchased from ABsciex (Framingham, MA, USA). A TripleTOF 5600 mass spectrometer from ABsciex and an HPLC system from Waters (Milford, MA, USA) were used.

For western blot, the primary antibodies for Alpha-1-antitrypsin (SERRINA1) (SERRINA1, ab9400), Ceruloplasmin (CP, ab51083), Transthyretin (TTR, ab9015), Apolipoprotein A-IV (APOA4, ab81616), Pro-epidermal growth factor (EGF, ab9695) and Prolactin-inducible protein (GCDFP15, ab62363) were purchased from Abcam (Cambridge, UK).

### Patients

In this study, 39 donors were recruited, including 23 diabetic patients and 16 healthy volunteers. According to the classification of American Diabetes Association [1], three groups diabetic patients were selected, including normoalbuminuria (urinary albumin excretion rates (UAER <20 mg/min, n = 7), microalbuminuria (20 ≤ UAER < 200 mg/min, n = 8) and macroalbuminuria (UAER ≥200 mg/min, n = 8). The clinical characteristics of all the donors were shown in Table 1 (detailed information in Additional file 1). The age, systolic blood pressure (SBP), diastolic blood pressure (DBP), body mass index (BMI), serum creatinine (Cr), UAER, estimated glomerular filtration rate (eGFR), triglycerides (TG), low-density lipoprotein (LDL) high-density lipoprotein (HDL), fasting blood glucose (FBG) and Hemoglobin A1C (HBA1C) among four groups were evaluated. The local ethical committee approved the protocol, and the diabetes patients were recruited from Peking Union Medical College Hospital. All subjects were informed about the purpose of the investigation and gave their written consent.

**Table 1:** Clinical characteristic of normal control, normalbuminuria, microalbuminuria and macroalbuminuria patients

Characteristics	Normal control (n = 16)	Normalbuminuria (group I) (n = 7)	Microalbuminuria (group II) (n = 8)	Macroalbuminuia (group III) (n = 8)
Gender (M/F)	8/8	3/4	5/3	4/4
Age (years)	47.25 ± 1.79	52.43 ± 2.91	47.33 ± 2.33	50.82 ± 1.7
BMI (mg/m^2^)	21.37 ± 0.43	25.77 ± 1.67^a^	25.28 ± 1.51^c^	24.39 ± 0.84
SCr (μmol/L)	76.85 ± 1.64	73.71 ± 4.13	84.78 ± 4.37	111.91 ± 4.31^e^
BUN (mmol/L)	5.16 ± 0.28	5.05 ± 0.59	4.78 ± 0.70	7.41 ± 0.41^e^
UAE (μg/min)	9.19 ± 0.96	6.81 ± 1.47	114.77 ± 17.63^d^	1489.2 ± 502.6^e^
eGFR (mL/min)	91.52 ± 2.62	90.66 ± 5.48	86.77 ± 5.54	57.58 ± 3.69^e^
TG (mmol/L)	0.88 ± 0.08	1.16 ± 0.14	2.03 ± 0.41^d^	1.58 ± 0.14^f^
SBP (mmHg)	119.33 ± 2.52	117.14 ± 5.96	123.89 ± 4.7	133.73 ± 4.41^f^
DBP (mmHg)	76.6 ± 1.64	74.29 ± 2.97	79.44 ± 4.12	80 ± 3.3
LDL (mmol/L)	3.31 ± 0.25	2.97 ± 0.27	3.27 ± 0.29	3.2 ± 0.24
HDL (mmol/L)	1.64 ± 0.06	1.31 ± 0.12	1.12 ± 0.1^d^	1.29 ± 0.08^f^
FBG (mmol/L)	5.01 ± 0.09	9.24 ± 1.17^b^	9.07 ± 0.58^d^	8.85 ± 0.64^e^
HBA1C (%)	n.a.	8.19 ± 0.62	8.76 ± 0.62	8.71 ± 0.44

*SCr* serum creatinine, *BUN* blood urea nitrogen, *UAE* urine albumin, *eGFR* estimated glomerular filtration rate, *TG* triglyceride, *SBP* systolic *blood* pressure, *DBP* diastolic blood pressure, *LDL* low-density lipoprotein, *HDL* high-density lipoprotein, *FBG* fasting blood glucose, *HBA1C* glycatedhaemoglobin

^a^ *P* < 0.05 between for normalbuminuria versus normal control

^b^ *P* < 0.001 for normalbuminuria versus normal control

^c^ *P* < 0.05 for microalbuminuria versus normal control

^d^ *P* < 0.001 for microalbuminuria versus normal control

^e^ *P* < 0.001 for macroalbuminuria versus normal control

^f^ *P* < 0.05 for macroalbuminuria versus normal control

### Urinary protein extraction

All the morning urinary samples from four groups were centrifuged at 5000*g* for 30 min, and the precipitates were removed. The supernatants were precipitated by 3 times volume ethanol for overnight at −4 °C. After 10,000*g* centrifugation for 30 min, the pellets were re-suspended in lysis buffer (7 M urea, 2 M thiourea, 0.1 M DTE, 50 mM Tris). The protein concentration of each sample was measured by Bradford method, and equal amount of total protein from each patients within each group were pooled together.

### N-linked glycoprotein enrichment

Each pooled sample was concentrated to above 6 mg/mL by vacuum centrifugal concentrator. After 1:1 dilution with buffer A (150 mM NaCl, 1 mM CaCl_2_, 1 mM MgCl_2_ and20 mM Tris, pH 7.4), each solution was incubated with ConA agarose overnight at 4 °C with rotation. After incubation, ConA beads were washed twice with buffer A and ConA-enriched urinary proteins were eluted by incubating the beads with 500 mM α-Me-d-Man in buffer A.

### Protein digestion and iTRAQ labeling

Each sample was digested using filter-aided sample preparation (FASP) method described in Wisniewsk et al. [37]. The proteins were reduced by 20 mM DTT at 37 °C for 1 h and were carboxyamidomethylated by 50 mM IAA at room temperature in dark for 45 min. Then, the samples were loaded onto 10 kDa ultrafilter tube (Pall, Port Washington, NY, USA), and were washed twice by 8 M urea. Next, the protein samples were further washed twice by 25 mM NH_4_HCO_3_. Lastly, trypsin resolved in 25 mM NH_4_HCO_3_ were added in protein samples, and digested the protein samples at 37 °C overnight. The digested peptides were collected as a filtrate. The normal and DN samples were individually labeled with 114, 115, 116 and 117 4-plexiTRAQ regents. Labeling was performed according to the manufacturer’s protocol (ABsciex). Finally the pooled samples were analyzed by 2DLC/MS/MS.

### Offline HPLC separation

The pooled mixture of iTRAQ labeled samples was fractionated using a high-pH RPLC column from Waters (4.6 mm × 250 mm, Xbridge C18, 3 μm). The samples were loaded onto the column in buffer A1 (H_2_O, pH = 10). The elution gradient was 5–25 % buffer B1 (90 % ACN, pH = 10; flow rate = 1 mL/min) for 60 min. The eluted peptides were collected at one fraction per minute. The dried 60 fractions were re-suspended by 0.1 % formic acid and pooled into 20 samples by combining fractions 1, 21, 41; 2, 22, 42; and so on. A total of 20 fractions from urinary peptide mixtures were analyzed by LC–MS/MS.

### Online LC/MS/MS analysis

Each fraction was analyzed with a reverse-phase-C18 self-packed capillary LC column (75 μm × 100 mm). The eluted gradient was 5–30 % buffer B2 (0.1 % formic acid, 99.9 % ACN; flow rate = 0.3 μL/min) for 40 min. A TripleTOF 5600 mass spectrometer was used to analyze eluted peptides from LC. The MS data were acquired using high-sensitivity mode with following parameters: 30 data-dependent MS/MS scans per full scan, full scans acquired at a resolution of 40,000 and MS/MS scans at a resolution of 20,000, rolling collision energy, charge state screening (including precursors with +2 to +4 charge state), dynamic exclusion (exclusion duration 15 s), MS/MS scan range of 100–1800 *m/z*, and scan time of 100 ms.

### Data processing

For database searching, All MS/MS samples were analyzed using Mascot (Matrix Science, London, UK; version 2.3.02). Mascot was set up to search the SwissProt human database (20,227 entries) assuming the digestion enzyme Trypsin. The parent and fragment ion mass tolerance was 0.050 Da. Carbamidomethyl of cysteine was specified as a fixed modification, and 2 mis-cleavage sites were allowed. Scaffold (version Scaffold_4.3.3, Proteome Software Inc., Portland, OR) was used to validate MS/MS based peptide and protein identifications. Protein identification was accepted at false discovery rate (FDR) less than 1.0 % on protein level and with at least 2 unique peptides. Proteins that contained similar peptides and could not be differentiated based on MS/MS analysis alone were grouped to satisfy the principles of parsimony. Scaffold Q+ was used to quantitate Label Based Quantification (iTRAQ, TMT, SILAC, etc.) peptide and protein identifications. Acquired intensities in the experiment were globally normalized across all runs. The reference channels were normalized to produce a 1:1 fold change. All normalization calculations were performed using medians to multiplicatively normalize data.

### GO and IPA analysis

All differential proteins identified were assigned a gene symbol using the Panther database (http://www.pantherdb.org/) comparing to the whole human urine proteome [38]. Protein classification was performed based on functional annotations using Gene Ontology (GO) for biological processes, molecular function and cellular component categories.

For IPA analysis, the SwissProt accession numbers were uploaded to IPA software (Ingenuity Systems, Mountain View, CA). This software categorizes gene products based on the location of the protein within cellular compartments and suggests possible biochemical, biological and molecular functions. The proteins were mapped to disease and function categories and canonical pathways available in the Ingenuity and other databases and ranked by z-score and *P* value respectively.

### Western blotting analysis

Western blot of individual samples was performed to validate proteomic quantitation of six selected candidate proteins, including SERRINA1, CP, TTR, APOA4, EGF and GCDFP15. Protein lysates were resolved on 6 % SDS-polyacrylamide gel, electro transferred to polyvinylidene fluoride (PVDF, Immobilon P, Millipore) membranes, and blocked in 5 % nonfat dry milk in Tris-buffered saline pH 7.5 (TBST, 100 mmol/L NaCl, 50 mmol/L Tris, 0.1 % Tween-20). Membranes were immunoblotted by primary antibodies against these candidate proteins, followed by secondary antibodies conjugated to horseradish peroxidase (HRP). The signals were detected by enhanced chemiluminescence (ECL, Pierce), and the chemiluminescence signals were recorded using a LAS 4000 system (ImageQuant LAS 4000 mini, General Electric Company, USA). The quantitative analysis of the resulting images was performed using Image J.

## Results

### Qualitative and quantitative analysis of urinary glycoproteome

Three group diabetic patients were included in this study, normoalbuminuria group, microalbuminuria group and macroalbuminuria group. The UAER increased gradually in microalbuminuria group and macroalbuminuria group; Scr and BUN increased while eGFR decreased significantly only in the macroalbuminuria group. These indicated that the renal damage aggravated increasingly in the three groups. There was no statically difference in other indexes among four groups.

**Fig. 2:**
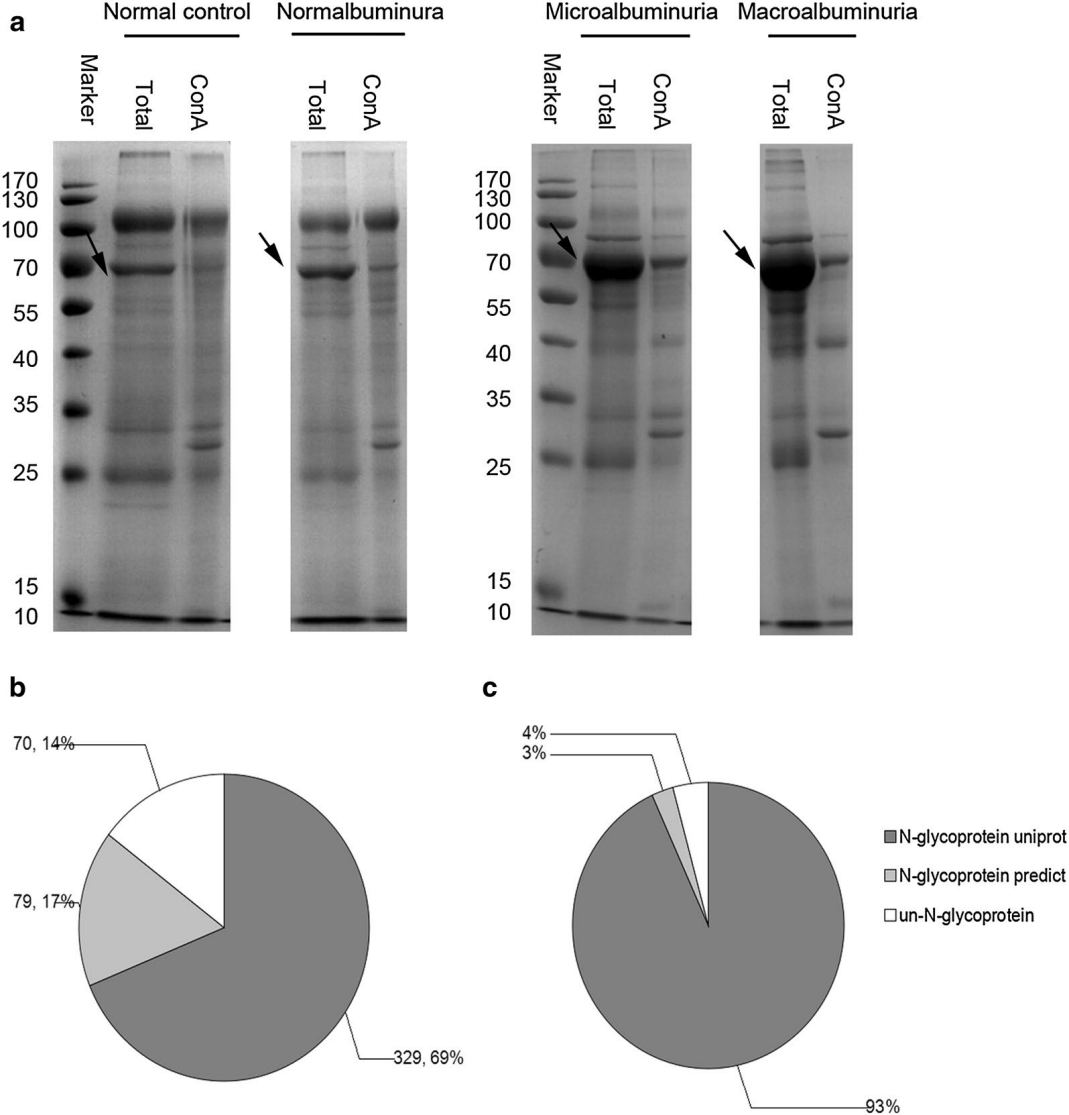
Qualitative and quantitative analysis of urinary glycoproteome. **a** SDS-PAGE analysis of glycoproteins enriched by ConA from different groups. *Total* total proteins, *ConA* ConA enriched N-linked glycoprotein. The *arrows* indicate albumin protein. Distribution of ConA enriched protein by quality (**b**) and quantity estimated by iBAQ (**c**)

N-linked glycoproteins from normal control, normalbuminura group, microalbuminuria group and macroalbuminura group were enriched by ConA lectin. Total urinary proteins and ConA enriched urinary glycoproteins were shown in Fig. 2a. Normal control and normalbuminuria had similar patterns, but the abundance of albumin increased in both microalbuminuira and macroalbuminuria group. After ConA enrichment, albumin band was dramatically decreased and some bands in other molecular weights (especially in 30KD) appeared in all the four groups.

By 2D-LC MS/MS analysis of ConA enriched samples, total 15,930 spectra and 2600 peptides from 478 proteins were identified (Additional file 2). By bioinformatic analysis, 329 proteins were annotated as N-linked glycoproteins in Swissprot database, and by NetNGlyc prediction [39] other 79 proteins were annotated as potential N-linked glycoproteins. Therefore, a total of 408 (83.6 %) ConA enriched proteins were N-linked glycoproteins (Fig. 2b). Intensity-based absolute quantification (iBAQ) could estimate the protein abundance in sample [40]. By iBAQ analysis, N-linked glycoproteins contributed 96 % abundance of total abundance (Fig. 2c, Additional file 2). The qualitative and quantitative analyses indicated the high efficiency of N-linked glycoprotein enrichment by ConA.

A total of 472 proteins could be quantified in all four groups (Additional file 3). By a ratio-fold change >2, (Table 2), 72, 107 and 123 differential proteins were found in normalbuminuria, microalbuminuria and macroalbuminuria (Additional file 4).

**Table 2:** Number of up-regulated proteins and down-regulated proteins in different stage of DN analyzed by LC–MS/MS

Stage	Normalbuminuria	Microalbuminuria	Macroalbuminuria
Up-regulation	34	62	74
Down-regulation	38	45	49

### Hierarchical clustering of differential proteins

**Fig. 3:**
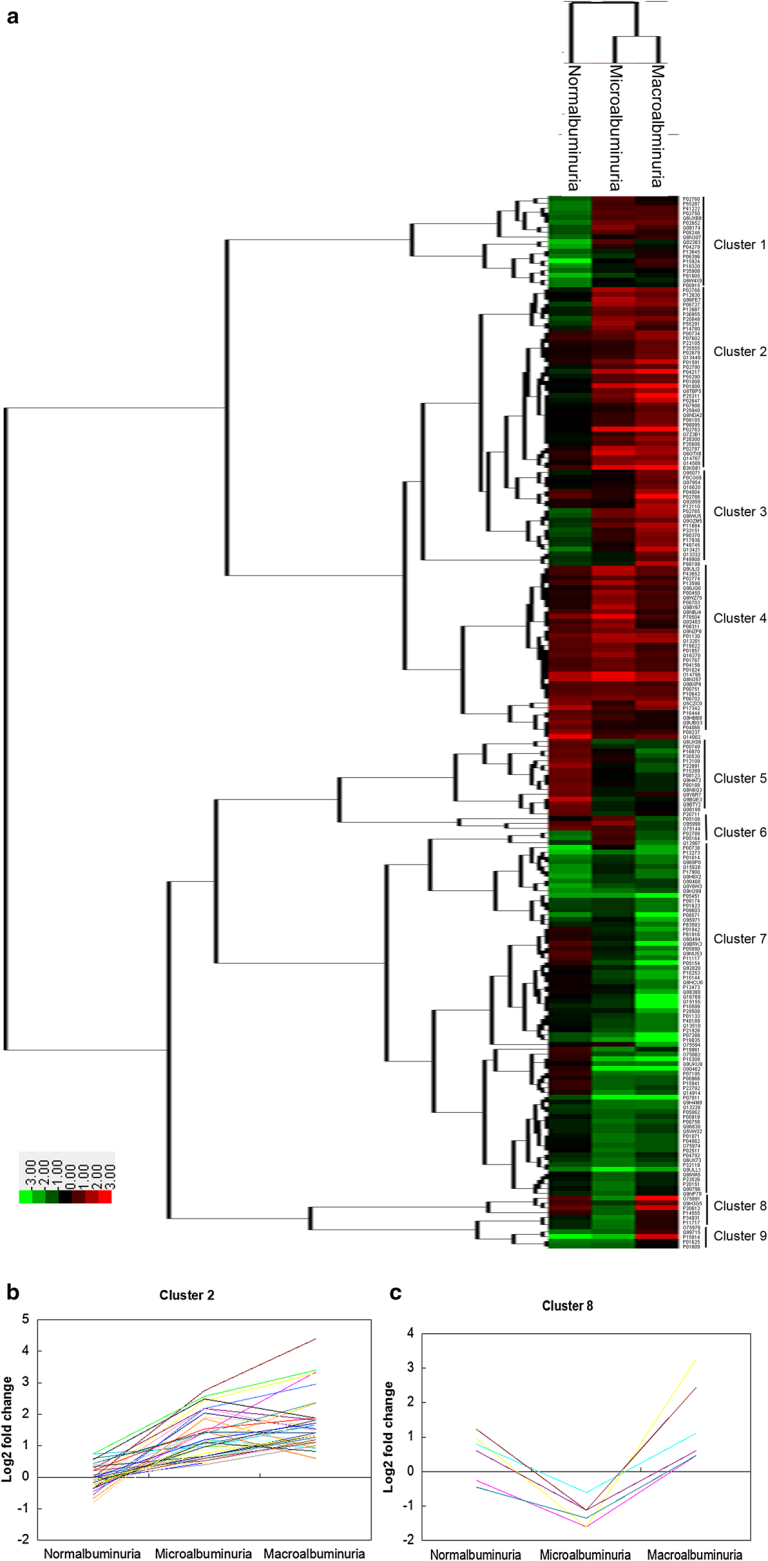
Hierarchical clustering analysis of the differential proteins in normalbuminuria, microalbuminuria and macroalbuminuria. **a** Hierarchical clustering analysis of the differential proteins. Protein fold change of cluster 2 (**b**) and cluster 8 (**c**) in normalbuminuria, microalbuminuria and macroalbuminuria

To get more information of differentially proteins during the course of DN, Hierarchical Clustering was performed by average linkage method. All the differential proteins were hierarchically clustered into nine clusters (Fig. 3a, detailed data in Additional file 4). In Cluster 2, proteins were overrepresented in both microalbuminuria and macroalbuminuria (Fig. 3b). These proteins might be leaked from the serum by glomerulus or secreted from the damaged kidney, and reflect the damage of the kidney during the course of DN. In Cluster 4, proteins were over-represented in all the three stages, which might reflect the pathological change of diabetes. In Cluster 7, proteins were underrepresented in both microalbuminuria and macroalbuminuria. These proteins might come from the health kidney and were down-regulated during the pathological processes of the DN. In Cluster 8 (Fig. 3c), proteins were down-regulated in microalbuminuria but up-regulated in macroalbuminuria. These proteins might reflect the early kidney impairment in microalbuminuria, but be enhanced by leaking from the serum in macroalbuminuria. Because the microalbuminuria was critical for early diagnosis of DN, the proteins in Cluster 2 and 8 may potentially be useful as novel biomarkers for early detection of DN [16].

### Functional analysis of differential proteins

**Fig. 4:**
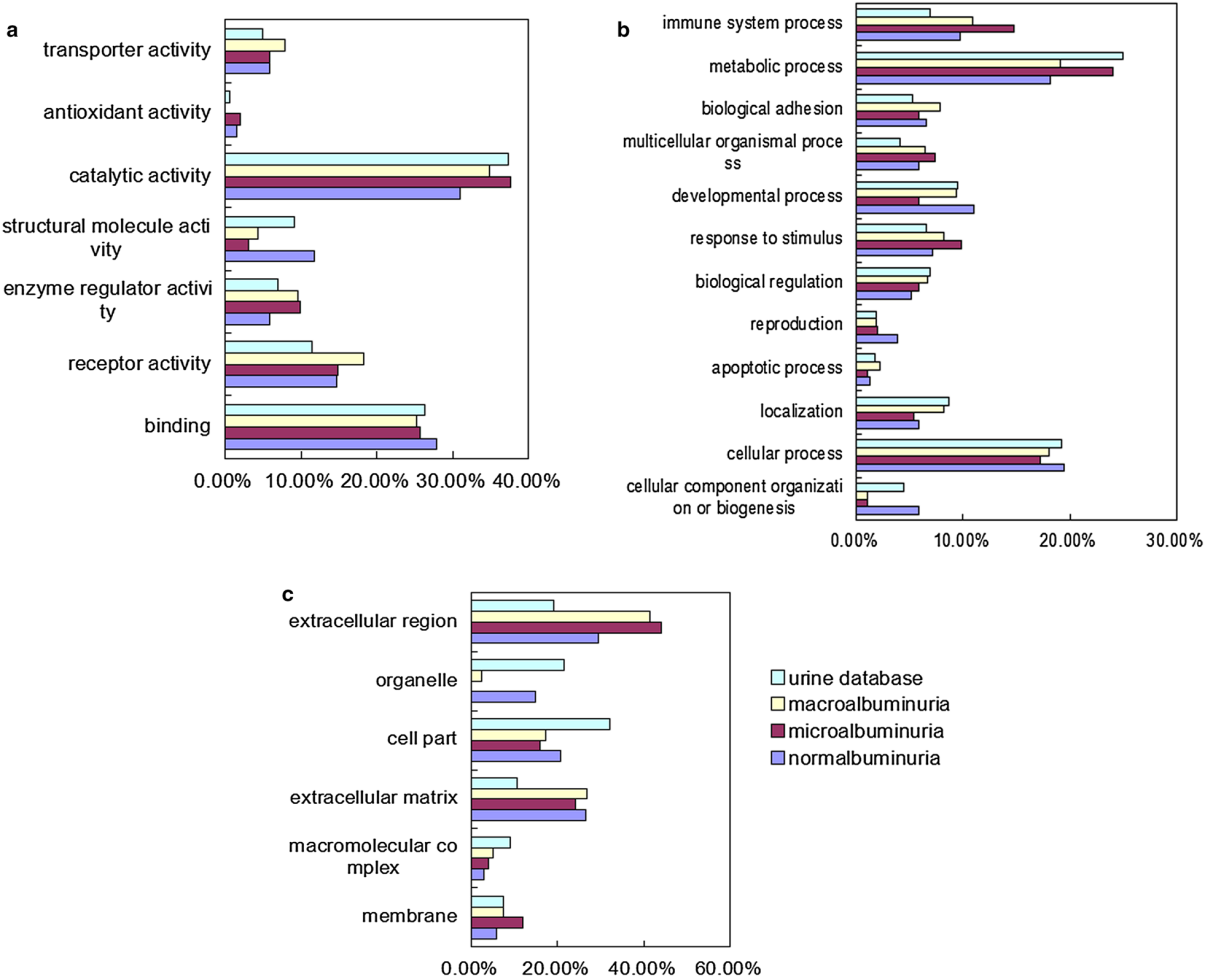
GO analysis of differential proteins during the course of DN. Differential proteins in normalbuminuia, microalbuminuria and macroalbuminuria were classified into molecular function (**a**), biological process (**b**), and cellular component (**c**) categories for human genes, comparing to the entire human normal urinary proteome by GO analysis. Categories with constitution of at least 2 % were displayed in the bar charts

The differential proteins were first analyzed by GO. The PANTHER classification system [41] was used to search the enrichment GO terms [38]. Differential proteins were classified into molecular function, biological process, and protein class categories. In the molecular function category, receptor activity and enzyme regulator activity were overrepresented, whereas structure molecular activity was underrepresented in DN (Fig. 4a). In biological process category, the terms of response to stimulus and immune system response were overrepresented, whereas the cellular component organization was underrepresented in DN (Fig. 4b). In cellular component category, extracellular protein was overrepresented, whereas intracellular protein was underrepresented in all the three stages (Fig. 4c). These results suggested that inflammation and immune system response were related to DN.

**Fig. 5:**
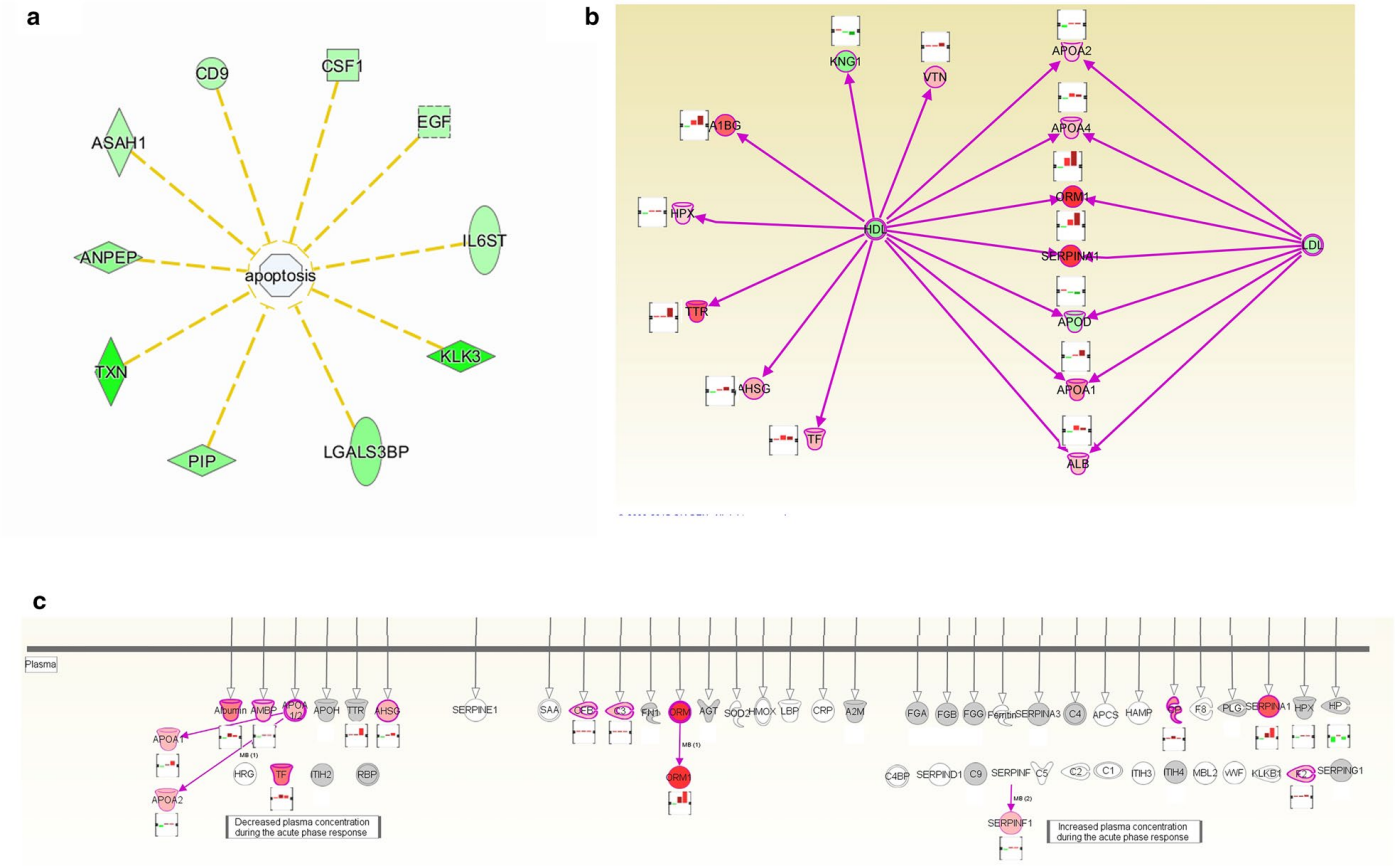
Relative functions and pathways in DN. **a** The apoptosis inhibitors were down-regulated in microalbuminuria. Differential proteins in HDL and LDL (**b**) and (**c**) acute phase signaling pathway in microalbuminuria (the protein expression changes in normalbuminuria, microalbuminuria and macroalbuminuria were shown in bar charts respectively)

**Fig. 6:**
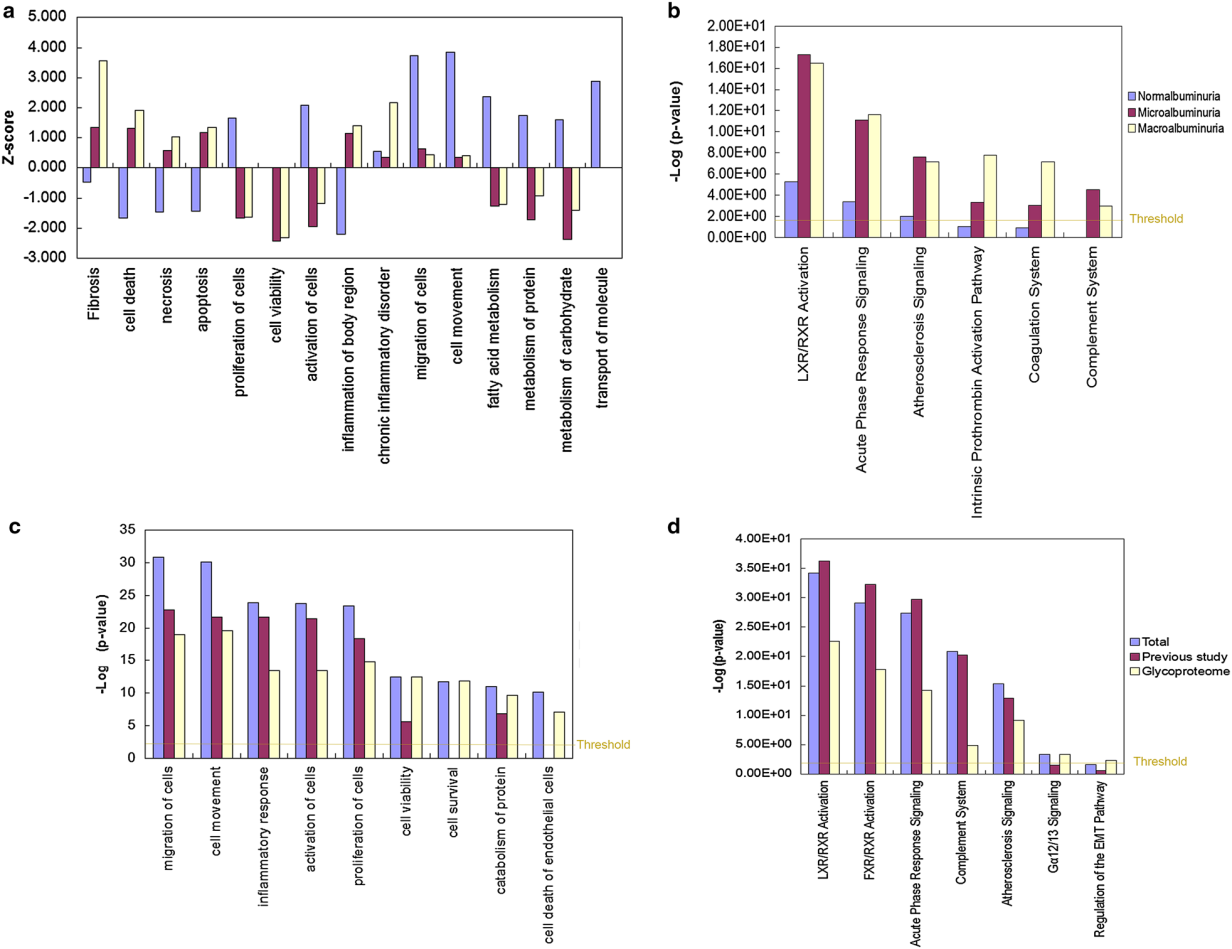
IPA analysis of differential proteins in DN. Function analysis (**a**) and top enriched pathways (**b**) in normalbuminuria, microalbuminuria and macroalbuminuria. Z-score >2: significantly activated; Z-score ≤2, significantly inhibited. −Log(*P* value) >1.5: significantly enriched. Function analysis (**c**) and top enriched pathways (**d**) in DN glyoproteomic study, previous study and the summarized of glycoproteomic study and previous study

To further analyze the detailed function change in DN, IPA analysis was performed. In disease and function analysis, in the normalbuminuria, cell proliferation, cellular movement and cell migration were activated; while cell death, necrosis, and inflammation response were inhibited, which might reflect the mesangial cell proliferation in the early stage of DN, and indicated the compensatory response of the kidney to resist the kidney damage caused by diabetes. Meanwhile, functions of carbohydrate, protein and fatty acid metabolism were activated, which reflect the high metabolism of the carbohydrate, fat and protein in diabetic patients [42]. In DN (microalbuminuria and macroalbuminuria), the functions of cell proliferation, cell viability were inhibited while the functions of cell death and apoptosis were activated (detailed data in Additional file 5). Several apoptosis inhibitors, such as EGF and GCDFP15, were down-regulated, indicating the activation of the necrosis of kidney cells, which indicated the discompensation status of DN (Fig. 5a). Activation of cell death and the inhibition of cell survival in urinary proteome were related to the cell death of renal cells in DN. Especially, in macroalbuminuria, inflammatory function and fibrosis were also activated, which reflected the inflammatory reaction and the kidney fibrosis process in the stage of macroalbuminuria. These reflect their reversible pathological change in this stage of DN (Fig. 6a).

To further detect the detail molecular mechanism of the metabolism abnormal and the inflammatory reaction in DN, pathway analysis was performed. LXR/RXR activation pathway, Acute Phase Response Signaling (APRS) pathway, and complementary pathway was remarkably enriched during the course of DN (Fig. 6b). 6, 16 and 16 differential proteins in normalbuminuria, microalbuminuria and macroalbuminuria were involved in LXR/RXR activation pathway. Many of them were mainly members of LDL and HDL, such as APOA4, SERPINA1 and TTR, indicating the abnormal of lipid metabolism increased during the course DN (Fig. 5b). Acute-phase proteins are a class of proteins whose plasma concentrations increase (positive acute-phase proteins) or decrease (negative acute-phase proteins) in response to inflammation. 5, 13 and 14 differential proteins in normalbuminuria, microalbuminuria and macroalbuminuria were involved in APRS (Fig. 5c). In microalbuminuria and macroalbuminuria, the downstream proteins of the APRS were up-regulated in urine, such as SERPINA1, CP and TTR. Elevated acute-phase proteins may reflect the inflammation and activation of innate immune system during the course of DN [43].

### Western blot validation

**Fig. 7:**
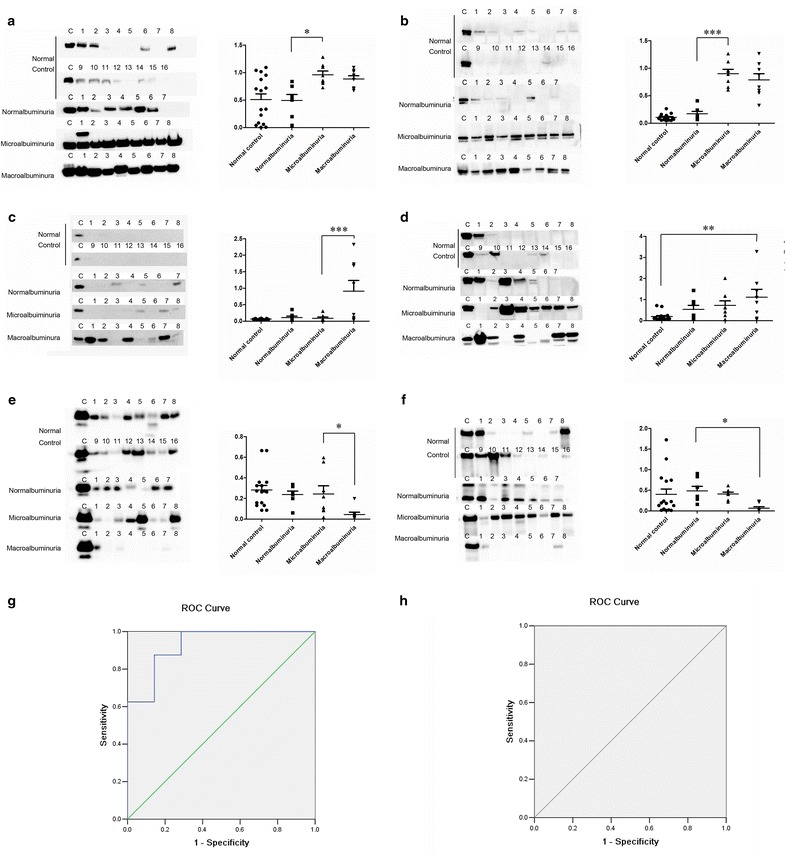
Western blot validation for six differential proteins. SERPINA1 (**a**), CP (**b**), TTR (**c**), APOA4 (**d**), EGF (**e**) and GCDFP15 (**f**). ROC curves for Western Blot validation of SERPINA1 (**g**) and CP (**h**) in microalbuminuric group versus normoalbuminuric group

By biological function and pathway analysis, six differential proteins, including SERPINA1, CP, TTR, APOA 4, EGF and GCDFP15 which involved in Acute Phase Response Signaling, cell death and apoptosis, or lipid metabolism were selected for Western Blot validation. As shown in Table 3, all six proteins had the similar trend to iTRAQ analysis by Western Blot. SERPINA1 (Fig. 7a) and CP (Fig. 7b) were significantly overrepresented in microalbuminuric and macroalbuminuric samples, comparing to normal control and normalbuminuric samples. TTR (Fig. 7c) was dramatically overrepresented in macroalbuminuric samples compared to the other three groups by more than tenfold. APOA4 (Fig. 7d) overrepresented progressively during DN process, and the difference between macroalbuminuric and normal control was significant. On the contrary, EGF (Fig. 7e) and GCDFP15 (Fig. 7f) were dramatically underrepresented in macroalbuminuric samples compared to other three groups.

**Table 3:** Selected six differential urinary proteins quantitative results in iTRAQ and Western blot analyses

Gene name	Accession number	iTRAQ quantification	Western blot
SERPINA1 isoform 1 of alpha-1-antitrypsin	P01009	1:0.94:5.37:9.84	1:0.97:1.88:1.73
CP ceruloplasmin	P00450	1:1.36:3.60:1.89	1:1.60:8.46:7.42
TTR Transthyretin	P02766	1:2.04:1.37:7.95	1:1.67:1.38:14.03
APOA4 apolipoprotein A-IV	P06727	1:0.58:3.73:3.15	1:2.70:3.67:5.67
EGF Pro-epidermal growth factor	P01133	1:0.89:0.65:0.38	1:1.20:1.01:0.16
GCDFP15 prolactin-inducible protein	P12273	1:0.21:0.39:0.25	1:0.84:0.86:0.16

Microalbuminuria stage is a very important stage for early diagnosis of DN. To evaluate the diagnosis effects of SERPINA1 and CP, which were significantly overrepresented in microalbuminuria, the ROC curves were plotted. Figure 7g, h showed ROC curves with high sensitivity and specificity to distinguish microalbuminuria and normalbuminuria. In ROC curve, Both CP and SERPINA1 had a good area under the curve value (AUC) (1.000 and 0.929, resp.). Because CP has an excellent AUC in our study, we further estimated the absolute concentrations of cut-off values for application. Narita et al. [44] measured the Ceruloplasmin excretion rate (ng/min) in normalbuminuirc patients and it ranged from 5.9 to 130 ng/min. Because in our study, all the CP levels of microalbuminuria patients higher than normalbuniuic, thus we suppose the cut-off values should be higher than 130 ng/min. But the accurate concentration range of CP in patients still need further work in large-scale clinical samples.

**Fig. 8:**
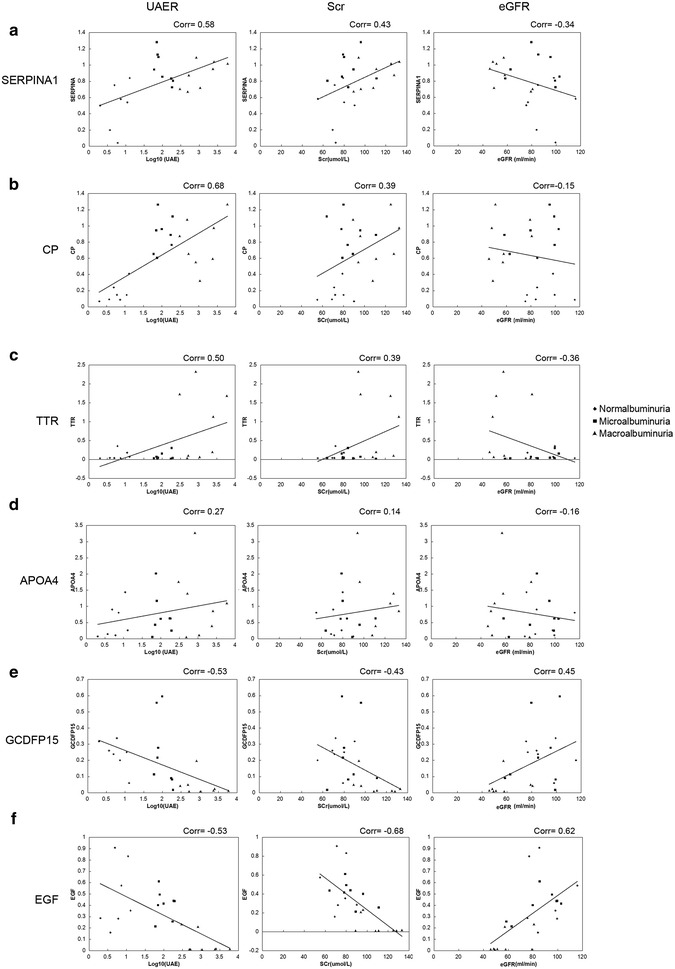
The correlation between the value of candidate biomarkers and individual urinary albumin excretion rates (UAER), estimated glomerular filtration rate (eGFR) and Serum Creatinine (Scr) values. **a** SERPINA1, **b** CP, **c** TTR, **d** APOA4, **e** GCDFP15, **f** EGF

We evaluate the relationship of the six new biomarkers to individual UAER, eGFR and Scr. As shown in Fig. 8, in the diabetic patients, the levels of UAER was positive correlated with the levels of SERPINA1, CP and TTR, negative correlated with GCDFP15 and EGF; the level of SCr was weak positive correlated with the levels of SERPINA1, CP and TTR, and negative correlated with the level of GCDFP15 and EGF. On the contrary, the eGFR was weak negative correlated with the levels of SERPINA1 and TTR, and positive correlated with the level of GCDFP15 and EGF. These indicated the five candidate biomarkers, including SERPINA1, CP, TTR, GCDFP15 and EGF, might related to the degree of the kidney injury by individual.

## Discussion

Previous urinary proteomics studies had discovered and validated several candidate biomarkers of DN, but the urinary glycoproteome change during the course of DN had not been studied. In our study, to better understand the disease status, we used ConA lectins to enrich urinary N-linked glycoproteome, and analyzed the samples with iTRAQ labeling and 2D-LC MS/MS. A total of 478 proteins were identified, including 408 glycoproteins, and 72, 107 and 123 differential proteins in normalbuminuria, microalbuminuria and macroalbuminuria were identified respectively.

By function and pathway analysis, we found that cell necrosis were inhibited and cell movement and cell proliferation were activated in DM, while on the contrary, cell survival, cell viability were inhibited while the functions of cell death and apoptosis were activated in DN, which might reflect the compensatory phase of kidney in the early stage of DN, and the de-compensatory phase of kidney in the late stage of DN. It has been reported that the high glucose induced the mesangial cell proliferation in DM, which was consistent with our finding in DN [45]. It has been reported that unbalanced apoptotic cell death leads to renal cell loss and has been observed in podocytes, tubular cells, and endothelial cells in experimental and human diabetic nephropathy, which was consistent with our result in DN [46]. Kidney fibrosis was also reflected in the macroalbuminuria group, which indicated the late stage pathological change in DN. By pathway analysis, we found a change in the members of HDL/LDL and acute phase proteins, which reflect the function abnormality in lipid metabolism and the inflammatory response during the course of DN, respectively. Six candidate biomarkers related to the above function and pathways were validated by Western blot in this study. As a prospective study would be desirable, a prospective study of another group patient to validate the potential biomarkers was also our future work.

SERPINA1 and CP were acute-phase proteins. SERPINA1 is a serine protease inhibitor, which target selastase as well as other proteases [12]. Neutrophil elastase degrades a range of substrates including elastin and other extracellular matrix proteins such as collagen, fibronectin, proteoglycan, complement receptors, thrombomodulin, lung surfactant, and several growth factors [47]. Therefore, in DN, the up-regulation of SERPINA1 would lead to inhibition of elastase and thus may contribute to accumulation of matrix molecules, and would maintain vascular elasticity and glomerular integrity. Previous study described that elastin was up-regulated in diabetic kidneys, which was consistent with the up-regulation of SERPINA1 in DN [48]. A previous study also reported the SEPRINA1 level was elevated in the kidney of microalbuminuria, and can cause matrix molecules to accumulate, which further approved that SEPRINA1 played a role in the pathology of DN [48]. CP is a metalloprotein that binds most of the copper in plasma and is involved in the peroxidation of Fe(II) transferrin to Fe(III) transferrin. The increase of CP in urine is caused by elevated intra-glomerular hydraulic pressure, which leads to the development of diabetic glomerulosclerosis [49–51]. The increase of SERPINA1 [12, 17, 52] and CP [52, 53] in the serum and urine of DN were identified in several previous studies and were validated by WB [12, 52], which were consistent with our results.

TTR and APOA4 were carrier proteins, and were components of HDL and LDL. TTR transports thyroid hormones in the plasma and cerebrospinal fluid, and also transports retinol (vitamin A) in the plasma. About 40 % of plasma TTR circulates in a tight complex with plasma retinol-binding protein (RBP) [17]. The transthyretin-RBP complex stabilizes the binding of retinol to RBP and decreases the glomerular filtration and renal catabolism of the relatively small RBP molecule. TTR was reported as a better and suitable marker for nutrition assessment in patients with chronic renal failure [54]. APOA4, like other apolipoprotein family, is a potent activator of lecithin-cholesterol acyltransferase in vitro. The mutations of Apolipoprotein A1/C3/A4/A5 gene cluster [55] and the ApoAI-CIII-AIV gene cluster [56] were associated with the lipid levels in type 2 diabetes mellitus and risk of coronary heart disease. Also, in the vitreous of diabetic macular edema, APOA-4 and other pigment epithelium-derived factor, like APOA-4, APOA-1, trip-11, and RBP were elevated [57]. Therefore, APOA4 and other apolipoprotein might participate in the pathogenesis of diabetes and diabetic complication. Previous studies have also shown that the TTR [13, 52] and APOA4 [17] increased in the urine of DN, which were consistent with our study, but no study had validated their changes. Our study firstly validated the up-regulation of TTR and APOA4 by WB.

Two underrepresented proteins, EGF and GCDFP15, reflected the inhibition of cell viability and the activation of cell death in kidney. EGF is an important growth factor which acts a potent mitogenic factor. It plays an important role in the growth, proliferation and differentiation of numerous cell types [58]. An experimental animal study [59] showed that the injection of EGF protein decreased the dilation of renal tubule, the apoptosis of renal tubular epithelial cells, and the atrophy of renal tubule and increases the proliferation of renal tubular epithelial cells. These results showed that the loss of EGF might play a role in the apoptosis of renal tubular epithelial cell in DN. GCDFP15 was a marker in breast cancer, and is required for the progression through G1 phase, mitosis, and cytokinesis in breast cancer cells [60]. However, no previous study identified or validated the change of EGF and GCDFP15 level in urine in DN patients. In our study, using proteomics method, we first identified the EGF and GCDFP15 decreased in the urine of macroalbuminuria DN, and validated the result by Western Blot. We speculated the down-regulated of GCDFP15 promoted the apoptosis of renal tubular or glomerulus cells in DN, but it needs to be further evaluated by experimental evidence.

**Fig. 9:**
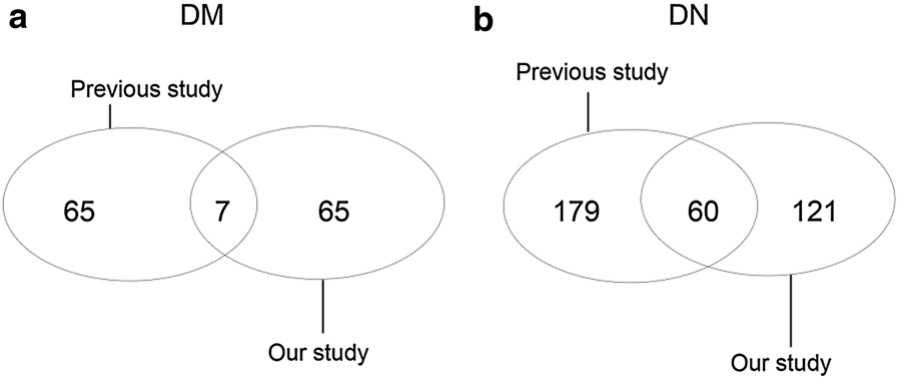
Differential proteins found in this study and previous studies in DM (**a**) and DN (**b**)

To better understand the patho-physiological change of DN, we summarized the differential proteins in diabetes and DN (microalbuminuria and macroalbuminuria) in previous study [14, 16, 18, 19, 28–31]. A total of 137 proteins (including 72 found in this study) and 361 proteins (including 181 found in this study) were changed in DM (Fig. 9a) and DN (Fig. 9b) respectively (detailed data in Additional file 6). Among the co-identified differential proteins, 6 of 7 and 46 of 60 were consistent with previous study in DM and DN.

**Fig. 10:**
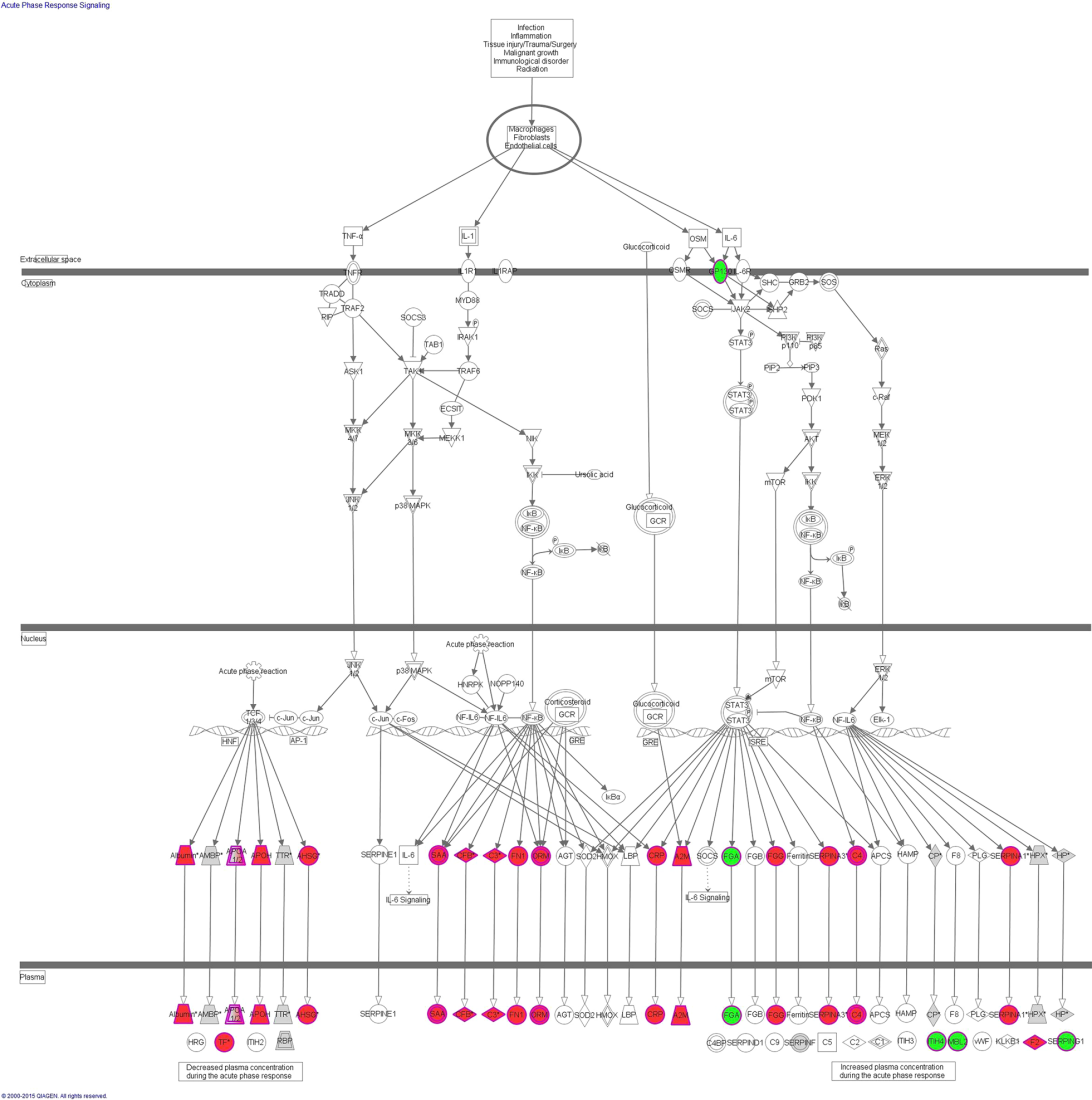
Differential proteins in acute phase signaling pathway in DN summarized in this and previous studies

IPA analysis was performed for all differential proteins in our study and previously studies. Functional annotation analysis of the differential urinary proteins showed that in DN, inflammation response, cell movement, cell migration, cell proliferation and cell death were enriched (Fig. 6c). By pathway analysis, LXR/RXR, FXR/RXR activation pathway, APRS, complement system was remarkably enriched in DN (Fig. 6d). In detail, 24 differential proteins in the urine of DM and DN were involved in LXR/RXR activation pathway. Many of them were mainly members of LDL and HDL. 25 differential proteins hit more than one third of the downstream proteins of Acute Phase Response Signaling (Fig. 10). The functional analysis of all differential proteins reflect the inflammation response, the abnormality of lipid metabolism, and the activation of the cell death in kidney cells in DN. Especially, the cell viability, cell survival, and cell death related proteins, and the regulation of EMT pathway were enriched only in glycoproteome study but not in previous convention proteomic studies (Fig. 6c, d). Therefore, urinary glycoproteome study could provide more cellular pathological and physiological information along the onset and progression of DN compared with the conventional proteomic studies.

## Conclusions

In this study, we analyzed the differential urinary glycoproteome during the course of DN. By function analysis, in normalbuminruia, cell proliferation and cell movement were activated, which reflect the compensatory phase. In micro- and macroalbuminuria, cell death and apoptosis was activated, which reflect the de-compensatory phase. By pathway analysis, urinary glycoproteome reflect the lipid metabolism abnormality and the inflammatory response in DN. The acute phase proteins and the member of HDL and LDL proteins were changed in DN; six candidate biomarkers were validated by WB, in which SERPINA1 and CP showed a high performance for early diagnosis of DN.

Our urinary glycoproteomic study identified some new differential proteins which could reflect cellular pathological functions, suggesting that urinary glycoproteome could provide more useful information of DN and could be applicable in the biomarker discovery of DN.

## Additional files

10.1186/s12967-015-0712-9 Detailed clinical characteristics of normal control, normalbuminuric, microalbuminuric and macroalbuminuric patients.
10.1186/s12967-015-0712-9 The iBAQ value and N-linked glycoprotein annotation of all identifications.
10.1186/s12967-015-0712-9 iTRAQ Quantitative protein and peptide list of urinary glycoproteome during the progression of DN.
10.1186/s12967-015-0712-9 Differential proteins in normalbuminuria, microalbuminuria, macroalbuminuria. The Cluster classification of each proteins were shown.
10.1186/s12967-015-0712-9 Detail protein list classified by biological functions by IPA in normalbuminuria, microalbuminuria and macroalbuminuria.
10.1186/s12967-015-0712-9 The differential protein lists in DM and DN from previous studies.

## Abbreviations

DN: diabetic nephropathy
ConA: concanavalin A
iTRAQ: isobaric tags for relative and absolute quantitation
HDL: high density lipoprotein
LDL: low density lipoprotein
AUC: area under curve
DM: diabetes mellitus
ESRD: end-stage kidney disease
2D-DIGE: two-dimension differential in-gel electrophoresis
CKD: chronic kidney disease
